# Factors influencing the provision of care for Inuit in a mainstream residential addiction rehabilitation centre in Southern Canada, an instrumental case study into cultural safety

**DOI:** 10.1186/s13011-021-00387-6

**Published:** 2021-06-29

**Authors:** Julie Lauzière, Christopher Fletcher, Isabelle Gaboury

**Affiliations:** 1 Département de médecine de famille et de médecine d’urgence, Université de Sherbrooke, 150, place Charles-Le Moyne, Longueuil, QC J4K 0A8 Canada; 2 Département de médecine sociale et préventive, Université Laval, 1050, avenue de la Médecine, Quebec, QC G1V 0A6 Canada

**Keywords:** Indigenous peoples, Inuit, Substance use treatment, Cultural safety, Cultural competence, Health equity, Health services research, Qualitative case study

## Abstract

**Background:**

Provision of culturally safe care has been proposed to address health inequity, including in the areas of mental health and addiction. The factors that influence the provision of culturally safe care remain understudied. This paper explores the factors influencing the efforts of a mainstream residential addiction rehabilitation centre to provide culturally appropriate and quality care for Inuit.

**Methods:**

An instrumental case study was conducted, informed by ethnographic and creative research methods. Over 700 h of participant observation were carried out between March 2018 and January 2020, in addition to qualitative semi-structured interviews (34 participants) and/or member-checking activities (17 participants) conducted with a total of 42 individuals: 20 Inuit residents, 18 clinical/specialized staff, and 4 clinical/administrative managers. An interpretive thematic analysis was performed to examine the factors that may influence the provision of culturally safe care for Inuit residents.

**Results:**

Ten categories of interrelated factors were identified and classified according to whether they relate to individual, programmatic, organizational, or systemic levels. These categories covered: (1) residents’ and staff’s life experiences; (2) personal and relational qualities and skills; (3) the model of care; (4) model flexibility; (5) ways in which relational aspects were considered; (6) sensitivity of the organization towards the population served; (7) human resources and professional development issues; (8) social climate; (9) political, relational, and funding climate; and (10) legislative, regulatory, and professional environment. While system-level factors generally had a negative effect on experiences of cultural safety, most factors at other levels had both favourable and unfavourable effects, depending on the context and dimensions examined.

**Conclusions:**

The results offer insight into the interplay between the challenges and barriers that mainstream organizations face when working with Inuit, and the opportunities and enablers that organizations can build on to improve their services. This paper contributes to a better understanding of the challenges and opportunities to providing culturally safe addiction programs to Inuit within a complex intervention setting. It concludes by highlighting some areas for improvement to advance cultural safety in this context.

## Background

The profoundly negative impacts of disempowerment, individual and collective suffering associated with colonial experience, and ongoing social, health and economic disparities are embodied in Indigenous people in many ways, including in high rates of substance use and destructive addictive behaviors [1–5]. Despite the clear need for culturally responsive addiction services in Northern Canada today, there are very few Inuit-specific or northern-based addiction services available. At present, when seeking relief from addictions, many Inuit find themselves in mainstream or First Nations addiction treatment centres in southern cities^2^. These environments are marked by a sometimes deep sociocultural, conceptual and linguistic distances, and power differentials between those providing services and the people receiving them. Failure to take these factors into account in the delivery of care risks perpetuating the inequities inherent in health systems by limiting access to care for Indigenous populations [6].

Provision of culturally safe care has been proposed as an avenue to address health disparities and to improve equity between Indigenous and non-Indigenous populations generally, and specifically in mental health and addiction fields [7–9]. Cultural safety refers to what people feel or experience when the care they receive recognizes, respects and nurtures their cultural identity, and safely meets their needs, expectations, and rights [10]. Cultural safety is a dynamic relational outcome that is defined by those who receive the service [7, 11]. A culturally safe experience is made possible through trustful and inclusive relationships, sincere commitment and dialogue, equitable partnership, and shared decision-making [12, 13]. For care providers and organizations, fostering cultural safety requires a continual process of self-reflection that permits self-awareness about the biases, attitudes, discourses, and practices that can influence their practice, and which are manifested as subtle to overt discriminatory events encoding the power imbalance between client and provider, indigenous and non-indigenous person [14, 15].

While the care experiences of Indigenous peoples have been examined through the lens of cultural safety [16–18], factors that influence the provision of culturally safe care are less well described in the literature. At the same time, having a better understanding of the challenges and opportunities faced by mainstream addiction rehabilitation organizations in their efforts to providing culturally appropriate and quality care to Indigenous populations could ultimately result in the design and implementation of more responsive and effective addiction services [19, 20]. Literature on mental health and addiction services points to a complex context with influences and interrelationships between factors at different levels [8, 19–22], which suggest the need to use an ecological perspective to understand factors that are likely to influence the translation of cultural safety into practice [23].

As part of a larger project that explores the contribution of cultural safety in identifying ways to improve the quality of and access to services offered to Inuit, this paper examines factors that may enable or limit the provision of culturally safe care for Inuit in a mainstream residential addiction rehabilitation centre.

## Methods

We conducted an instrumental single case study [24], as this qualitative study design allows for an in-depth, holistic understanding of complex phenomena within the context of a given real-life bounded system [25, 26]. It was expected that a single case study design would allow for a comprehensive and nuanced understanding of social interactions in context, and thus of cultural safety for Inuit in a selected care setting. To our knowledge, this is the first study to use the concept of cultural safety within a case study design to generate rich, contextualized data on the factors influencing the efforts of a mainstream residential addiction rehabilitation centre to provide culturally appropriate and quality care for Inuit. To maximize what we can learn from the case, we purposively selected an addiction treatment centre that serves a large number of Inuit as only they can determine whether their care experiences are culturally safe or not [7, 24].

### Study setting

The selected case was a well-established private institution in the southern region of the province of Quebec, Canada. The institution offers mainstream outpatient and residential addiction rehabilitation programs, aftercare and continuing care programs, supervised apartments, employment reintegration services, and family support services. Main services are oriented to individuals with substance use problems, with different residential programs tailored for specific categories of program users. Individuals could self-refer or be referred to programs by a variety of sources; among them family members, social services, health services, or the courts. All programs are voluntary and available free of charge or at a minimal cost to Quebec residents. Since its inception, the centre has worked with individuals from various ethnic and cultural backgrounds, including Inuit, who represented less than 5 % of residential program users.

The centre works with a therapeutic community approach, which is based on mutual help [27]. Programs offered reflect best practices in substance use rehabilitation such as motivational; cognitive-behavioral relapse prevention; and community reinforcement approaches. Program users support each other to develop a set of skills to promote a healthy lifestyle free of alcohol and drugs. Skills developed during programs include expressing oneself appropriately and being able to ask for help, to resolve conflicts and to support peers. The main role of the staff is to ensure the therapeutic quality of the care environment. Residential programs last approximately 6 months, with new residents being admitted weekly. Residents progress in gender-specific, highly structured, five-phase programs. Over the course of their program, residents assume greater personal and social responsibilities in their therapeutic community and eventually become role models to new residents.

### Data collection and participants

Data was collected by the first author (JL) over a two-year period through participant observation and in-depth interviews, followed by member-checking activities. The centre’s website and annual reports were also reviewed to better understand the setting and to contextualize data.

### Participant observation

From March 2018 to January 2020, 36 participant observation visits were made, ranging from one hour to five days, for a total of 67 days and 31 overnight stays on site. Most visits occurred in two geographically separate facilities where the first author (JL) shared the daily lives of Inuit women residents. At each visit, she introduced herself and the study to everyone she met and made sure that they consented to her presence. Participant observation allowed for close observation of purposively selected activities and interpersonal interactions, including different types of clinical, sociocultural, and administrative activities. Data was recorded on site or soon after the observation periods and field notes helped cover contexts, activities, actors, interactions, informal discussions, and resources.

### Interviews and member-checking activities

From March 2018 to March 2019, in-depth interviews were conducted with a total of 34 participants, including 15 Inuit residents, 16 clinical/specialized staff, and three clinical/administrative managers. Individuals were purposively invited to participate in interviews to maximize diversity based on age, sex/gender, and life experiences (Inuit participants) or based on their role and the intensity of their contact with Inuit residents (staff, managers). Following an informed consent process, participants were interviewed using semi-structured, open-ended interview guides covering participants’ respective experiences and interpersonal relationships, as well as perceived barriers and enabling factors influencing Inuit access, engagement, and retention in the centre programs. Interviews were conducted in closed rooms in the centre’s premises, in their preferred language (French or English). Median duration of interviews was 60 and 59 min respectively for Inuit residents and staff/manager participants. Audio-recorded interviews were transcribed verbatim, then checked for accuracy. For participants who did not consent to audio-recording, minimal notes taken during the interview were subsequently expanded.

From September 2019 to January 2020, the first author (JL) organized member-checking activities to share and discuss her preliminary interpretations with a total of 17 purposively selected individuals (8 Inuit residents, 8 staff, and 1 manager), nine of whom formerly participated in in-depth interviews (3 Inuit residents and 6 staff). Member-checking activities were either game-based, small group discussions (Inuit residents) or visual model-based elicitation discussions (staff, manager).

Overall, 20 Inuit residents, 18 staff, and 4 managers participated in interviews and/or member-checking activities (Table 1). In the year prior their admission to the program, most Inuit participants were using alcohol and to a lesser extent cannabis; only a few were using other drugs (e.g., cocaine, hallucinogens, opioids). All staff and managers were non-Indigenous with varying work experiences.

**Table 1:** Participant characteristics^a^

Characteristic	N (%) or median (range)	
	**Inuit residents (*n* = 20)**	**Staff, managers (*n* = 22)**
Age (years)	32 (18–61)^b^	41 (24–67)
Sex (women)	19 (95)	15 (68)
Ethnicity (Inuit)	20 (100)	0 (0)
Lifetime personal experience with problematic substance use	20 (100)	15 (68)
Months in residential program^c^	3.8 (1.0–6.7)	–
Previously participated in any residential addiction treatment program (yes)	13 (72)^d^	–
Work experience		
Years in current job	–	5.7 (0.5–23.0)
Years in addiction treatment	–	8.8 (1.0–33.0)

^a^Data from sociodemographic questionnaires. ^b^*n*=16. ^c^At the first interview. ^d^*n*=18

### Data analysis

This paper draws primarily on the perspectives of staff members and managers, although data from interviews with Inuit residents and participant observation also informed the iterative analysis process [11]. The perspectives of Inuit residents were used to determine what was deemed culturally safe or not to them, and then we built on this understanding to identify and discuss facilitators and barriers to providing culturally safe care.

We performed an interpretive thematic analysis [28]. Following an immersion in data, the first and third authors (JL and IG) met to identify emergent themes and to form an initial coding scheme from a selection of transcripts covering all participant categories and interview types. The first author (JL) coded all transcripts using NVivo and refined the initial coding scheme to further identify the factors influencing the provision of culturally safe care for Inuit.

Emerging factors were then organized using a multi-level framework which posits five levels of analysis: structure, organization, patient, provider and innovation [29]. We chose this implementation framework because it considers ‘patient’-related factors, which is consistent with the idea that they are the ones who can determine if the care they receive are culturally safe for them. Triangulation of interviews and observational data was used to enhance validity of findings [24]. Preliminary findings were discussed with participants during member-checking activities [24, 28]. In the final stages, we merged the ‘patient’- and provider-level factors into a single category of individual-level factors. Throughout the research process, regular team discussions helped to enhance the analytical process of the study.

## Results

**Fig. 1:**
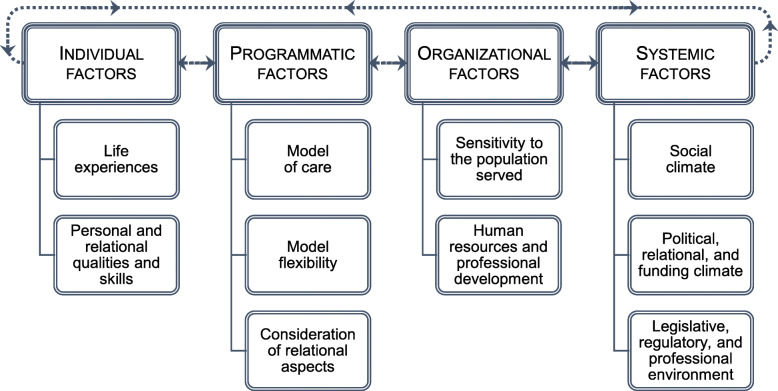
Factors that may influence the provision of culturally safe programs for Inuit residents

The analyses identified ten categories of interrelated factors that may influence the provision of culturally safe programs for Inuit residents. They are reported according to whether they relate to individual, programmatic, organizational, or systemic levels (Fig. 1). All quotes included in the paper are from staff members or managers (collectively referred to as “staff” hereafter), identified by pseudonyms. Most have been translated from French, the language in which they were collected.

### Individual factors

Individual-level factors refer to elements that may have influenced the relational dynamics with and within the programs. They were related to the level of relational comfort, the development of trust and/or the way in which members of the therapeutic community relate to each other. For Inuit residents, these factors may also have influenced their needs or expectations of the program and their assessment of the care they received, including whether or not they found that it was culturally safe for them. Two predominant categories of individual factors were identified: (1) life experiences; and (2) personal and relational qualities and skills.

#### Life experiences

Inuit residents generally felt that they shared a similar set of life experiences, which were quite different from those of non-Inuit residents and staff. Moreover, a greater or lesser sociocultural distance existed between individuals depending on their respective life experiences. Increased sociocultural distance was often associated with a greater lack of knowledge, culture or value shock, misjudgments or misinterpretations, mistrust, closedness, and resistance; most of these elements were observable in Inuit and non-Inuit alike.

For most Inuit residents who live in the North, their relocation to a mainstream program in Southern Quebec was destabilizing and contributed to their disorientation at the beginning of the program. Some Inuit residents, however, had experiences of living in the South or of sustained relationships with non-Inuit. Inuit residents’ prior relationships with non-Inuit affected their perceptions of and relationships with staff and other residents in the program. Negative experiences, particularly within the child welfare or justice systems, were likely to fuel judgments and mistrust towards ‘White’ people. Conversely, positive experiences with non-Inuit and a greater familiarity with the ways of doing in the South generally contributed to lessening the culture shock and mistrust that Inuit residents could have at the beginning of their program, facilitating their integration into the resident community and their participation in the program.

Each Inuit resident had their own assemblage of connections to Inuit values and cultural practices, such as family; Inuit language (Inuktitut), food, and traditional activities; and spirituality and religion. This influenced the needs of Inuit residents to have these represented in their living environment, and thus in programs. Some staff reported that historical trauma experienced by Inuit could explain why some people had a complex relationship with or even no apparent connection with Inuit culture and traditions. Individual experiences of trauma, including physical, sexual, and emotional violence and/or the sudden or violent death of loved ones, also influenced the interpersonal relationships of Inuit residents with other members of the therapeutic community. That the life stories of many Inuit residents were marked by violence, anger, and aggressivity was sometimes interpreted by other community members as traits of Inuit or of Inuit culture rather than associated with the difficulties they experienced.

Among staff members and non-Inuit residents, few had ever had a relationship with Inuit (or Indigenous people) prior to their encounters in the program. Many non-Inuit were unaware or confused about diversity among Indigenous Peoples, including of differences that exist between First Nations and Inuit. Most non-Inuit had a limited knowledge of Inuit history, values, ways of being and doing, and of realities specific to Inuit or Northern contexts. As a result, direct or indirect exposure to elements such as Inuit ways to communicate, traditional foods, customary adoption, and cost of living, at times elicited mixed reactions from non-Inuit, making Inuit residents feel misunderstood or judged. For the staff and non-Inuit residents who indicated they had prior contact with and knowledge of Inuit (or Indigenous people generally), these had generally taken place in contexts or situations likely to feed a negative or stereotypical image of them (e.g., homelessness, detention, addictions).

Staff members and non-Inuit residents had varied life paths, some of which had similarities with the experiences of Inuit residents. Some of the staff members came from backgrounds of problematic substance use themselves and some were former residents of the participating centre. Many of the non-Inuit residents and some staff also had experiences that were described as difficult and marked by violence, poverty, and impacts of their alcohol or drug use on their lives. This helped them to understand their respective realities and thus fostered the development of relationships.

“I too come from a rough background, you know, while I was drinking. So I was able to understand them; I know what the rush of the street is as well as living in violence and being in difficulty and poverty and everything. So I think it brought me closer to them, from the beginning.” (Mélanie).

Coming from “another culture” or having faced discrimination in their personal lives also led some staff and non-Inuit residents to be more sensitive and empathetic to the situation of Inuit residents in the program.

#### Personal and relational qualities and skills

While life experiences influenced the sociocultural distance between people, personal and relational qualities and skills – especially those of staff – brought members of the therapeutic community closer together. Staff participants were curious about the culture and realities of Inuit residents and interested in getting to know and understand them better. This interest led staff to ask questions and to work in ways that fostered dialogue and relationships. Staff saw this as requiring time, openness, patience, and consistency on the part of everyone involved. According to staff, other critical elements to mitigate the sociocultural distance and to work effectively with Inuit included respect, listening, empathy, humility, as well as emotional involvement and willingness to step out of one’s ‘comfort zone.’

“It opens your mind to having to really wipe your slate clean in your mind as to everything you’ve learned already, or that you are, because you’re White and you live in the [South]. And then it forces you to acknowledge and really ask and really observe and really listen to what they’re saying. Because it’s not always what you’re reading.” (Caroline).

The language barrier was important in the relational dynamic of the therapeutic community. A good command of English was often necessary to connect with Inuit residents for whom English was often a second language and which they had mastered with varying fluency. Likewise, being aware of and adjusting one’s ways of being and communicating was valuable in interactions with Inuit residents. The tone and rhythm of communication, especially the level of comfort with silence, and attention to non-verbal language were emphasized as key elements. In addition, staff who had more experience working with Inuit residents often used humor to facilitate communication with them.

The dynamics of interpersonal affect and language proficiencies shaped the willingness and comfort of Inuit to answer the questions of non-Inuit and their openness to discuss their lived-realities and cultural ways.

### Programmatic factors

Program-level factors refer to elements that may have influenced, on the one hand, the relational dynamics with and within the programs, the level of comfort of Inuit residents, and their assessment of the care they were receiving; and on the other hand, the fittingness of programs to meet Inuit residents’ expectations, preferences, and needs. Three predominant categories of programmatic factors emerged from the analyses: (1) the model of care; (2) its flexibility; and (3) the consideration of relational aspects.

#### The model of care

 As a person-centred model of care, the therapeutic community approach was perceived by staff as a fertile ground for interactions that were respectful of people and their culture. Thus, although the concept of cultural safety was new to participants at the beginning of the study, staff felt that their programs were conducive to it.

“I don’t think that we pay much attention to cultural safety… Well, not specifically for Inuit. But I think that just as we deal with all other program users, we stop at who you are, where you come from, what is important to you and then we reinforce it. So indirectly, we do.” (Marie-Josée).

Staff perceived that the therapeutic community approach, being based on mutual help, was globally relevant and appropriate to be used with Inuit since staff saw the Inuit as being part of a collectivist society. That said, there seemed to be different interpretations of what mutual help means, and the way it manifests on a daily basis. For instance, sharing food or other necessities was a central aspect of mutual help for Inuit residents, whereas for therapeutic purposes staff discouraged this type of sharing within the programs; staff valued peer support aimed at changing addiction-related behaviours more highly.

While all programs were designed to help achieve and maintain sobriety, many residents were still ambivalent about quitting drinking or using altogether. Staff participants felt that the goal of sobriety could be especially ambitious for Inuit considering the extent of alcohol and drug use in the North. This observation sometimes prompted staff and other residents to adopt an overprotective approach towards Inuit residents that made them feel misunderstood, judged, or mistrusted. Aiming at sobriety also seemed to be out of step with the harm reduction approach favoured by the social services in the North.

Most Inuit residents did not have a clear idea of what they were getting into when they came to the centre. Their reasons for coming were varied but were often associated with the fact that the programs were offered (partly) in English and were long-term. Many Inuit residents felt they needed a program longer than few weeks, often after relapsing following a short program and/or because court conditions imposed on them required a long program. Many Inuit women also chose the centre because of its Mother and Child Program.

As for program modalities, some were perceived by the staff and Inuit residents as incongruent with Inuit culture and realities. The program is based on ways of being and interacting that were different from the ones most Inuit residents were used to. These incongruities could amplify discomfort of Inuit residents in the program and their feelings of being misunderstood, judged or not respected. The structure and sustained rhythm of the days, the predominance of group rather than individual activities and interventions, the choice of therapeutic tools used, and the unavoidable use of English or French to interact in the program also appeared to fall short of expectations or preferences of a number of Inuit residents and to limit or slow down their participation in the program.

#### Model flexibility

Flexibility of the therapeutic community model was, however, very much in evidence in the comments of staff participants. Although recognized as having a highly structured approach, staff emphasized the many changes made over time to better meet the needs of their program users. A number of accommodations were made to facilitate admission and integration of Inuit in the residential programs and/or to improve their retention and program completion. For example, although future residents were expected to arrive sober and by their own means, the centre took care of the transport between the airport and the centre for Inuit coming from the North (if not paid/organized otherwise) and they could be admitted even if they had consumed alcohol on the plane. This accommodation was put in place to avoid Inuit finding themselves in a vulnerable situation, intoxicated and with no place to go in a city they knew little if at all. Other examples of accommodations included the implementation of video calls to allow Inuit residents to stay in touch with their relatives living in the North as they could not visit as often as most other residents, and instances when Inuit residents of different genders were permitted to meet on occasion (thus breaking the separation of the genders in programs) to facilitate their integration. Finally, Inuit residents were allowed to make longer home visits than most other residents. But as transportation costs to the North are very high, it was rare for Inuit to make more than one home visit over the course of their program.

The centre also organized occasional activities for Inuit, where the atmosphere was closer to what was familiar to them. About once a month, these gatherings allowed them to spend time outside of the program structure, as well as to connect with Inuit ways in that they could speak Inuktitut and eat traditional foods. They also met quarterly with visiting Inuit Elders. Otherwise, staff also encouraged Inuit residents to share their flavour of life with the therapeutic communities, for example by using Inuktitut to pray in the community morning meeting or to perform throat signing on special occasions or events. For staff, making room for Inuit cultural practices in programs represented a way of showing respect towards Inuit residents, acknowledging their unique ways, and contributing to their well-being.

“You have to make people as happy as possible. And it’s often by presenting components of their identity that people will say: ‘ah, you know me well. I’m going to trust you… You know what I mean… You know how things are at home’ […] If we improve [programs] with cultural elements for the care and for the benefit of the program user, I’m all in.” (Paul).

Staff were confident in the ability of their model of care to support Inuit in their recovery, but they emphasized that more work needed to be done to better tailor the program modalities to the Inuit context. That some adaptations at times did not go smoothly or had temporarily accentuated the cleavage between the Inuit and non-Inuit worlds was perceived by staff as part of a normal learning process under the circumstances. Staff also stressed the importance of establishing a dialogue between Inuit and non-Inuit to arrive at appropriate and effective programs where Inuit residents feel comfortable, valued, and respected. For staff, the main limitations to the flexibility of the model of care were related to its integrity, as well as the legislative, regulatory, and professional frameworks to which the centre and programs had to conform.

#### Consideration of relational aspects

Staff repeatedly emphasized both the difficulty and the importance of building and maintaining trust with Inuit residents. This concern was present even before their program admission. As most Inuit did not have the chance to visit the centre prior to their admission, efforts were made by admission staff to maintain a regular telephone contact with them until their arrival. On site, the long program duration coupled with the environment conducive to informal conversation offered multiple opportunities to develop relationships among the therapeutic community members. As much as possible, new Inuit residents were paired with Inuit mentors (i.e., more advanced residents in the program) and with staff members who had more experience working with Inuit. Relational continuity was favoured by staff, although it was limited by staff and residents turnover and occasional mobility between programs. Furthermore, clinical and administrative workload of staff was a factor limiting the time available to develop trustful relationships with residents.

To lessen the culture shock and facilitate the integration of Inuit residents, staff tried their best to have more than one Inuk at once in programs. Having Inuit peers was highly appreciated by Inuit residents as they felt better understood by them. For staff, the greatest benefits to the presence of Inuit peers manifested when they were distributed in the different phases of the programs, so that more advanced Inuit residents could help new ones in their program.

Although programs were said to be bilingual, translation from French to English was not readily available in all activities, especially in informal conversations. Having to ask repeatedly for translation was a major irritant for Inuit residents who needed it and could make them feel not respected or ignored. Conversely, the presence of Anglophone residents in otherwise mainly Francophone programs generally contributed to a better balance in the resident community. While Inuit residents could use Inuktitut among themselves during their program, there were various situations where they were asked to switch to English or French to express themselves. These were usually situations where other community members wanted to understand what they were saying so that they could benefit from their perspective or offer them support. Occasionally, however, the request was for Inuit residents’ conversations with their relatives, which made it even more disturbing to Inuit. Such situations were considered deplorable by staff but nevertheless inevitable because of the nature of the model of care, that is mainly based on residents’ mutual help.

 Although sought out and seen as enriching and essential to the model of care, the diverse profiles of the therapeutic community members brought challenges in terms of managing cross-cultural relations within the therapeutic environment. Inuit represented the largest racialized group in the visited programs. While there were advantages to having more than one Inuk resident per program, staff reported that it could become problematic when they tended to group together to the point of not letting other members of the therapeutic community enter their circle. Tensions increased, for example, when staff or other residents insisted that Inuit residents limit their contact with each other and mix more with other residents. At other times, the activities specially organized for Inuit residents aroused envy or jealousy on the part of some non-Inuit residents who perceived these activities as enjoyable moments or privileges reserved for Inuit instead of therapeutic activities. Interventions by staff to put things into perspective and analyzing situations holistically generally helped bring people together in such circumstances.

Staff also placed a great deal of emphasis on the commonalities between the residents, particularly with respect to addiction and recovery, to foster cohesion and the development of relationships between them. During the program, Inuit residents were often made to share their housing units with non-Inuit residents. Whereas this strategy could be more demanding in terms of cultural adaptation, it was perceived by staff as being safer in the medium term because it encouraged fraternization.

“In the beginning I was wondering about the fact that, for example, Inuit residents are put in mixed units [along with non-Inuit residents], that there is not one unit that is only Inuit. Because I was thinking, by putting them all together, they will create a way of life, if you will, that resembles their own living environment. And I realize that probably if we had done that or if we were doing it now, we would be moving away from a safer practice, where I think we can all sing Happy Birthday in Inuktitut. […] It makes it so that these common bridges are created on things that are similar, […] I think there is something reassuring in that.” (Sylvie).

### Organizational factors

Organization-level factors refer to elements that may have influenced how the organization was mobilizing different resources to deliver culturally safe programs for Inuit. The two predominant categories of factors identified are: (1) sensitivity to the population served; and (2) human resources and professional development.

#### Sensitivity to the population served

Over time, the centre has welcomed a growing number of Indigenous peoples, raising awareness of the centre staff, managers, and administrators about Indigenous needs and challenges with regards to recovery and healing. The experience thus acquired with First Nation members and Inuit over 30 and 10 years respectively, has contributed to the centre’s increased openness to adapt its activities and programs to better serve Indigenous residents. These various experiences have contributed to improving the centre’s general understanding of Indigenous issues and to developing greater humility among the staff – particularly those who have been working at the centre for longer periods – with respect to their contribution to solve/mitigate addiction in Indigenous communities.

“I think that more and more, we are offering cultural safety [to Inuit residents]. In the sense that we respect them a lot more, we understand them more. […] Are we perfect? No. I think we still have a lot to gain. But I think we are much better. We are much humbler than before. And I think we are much more open, too, to moving forward with all that.” (Sarah).

In addition to various accommodations and activities for Inuit residents, the centre has developed other special projects to support Inuit and improve continuity of care for them. Thus, starting in 2013, some of the centre staff have been holding weekly group meetings and on-demand individual meetings with Inuit inmates in two provincial correctional facilities. These activities facilitated the referral of inmates interested in undertaking a more comprehensive approach to the centre’s programs where they could deepen the work begun in the meetings while in detention (depending on the inmates’ needs, some are rather referred to other programs). Starting in 2018, a few staff have been visiting selected villages in Northern Quebec approximately monthly to offer aftercare support to former Inuit residents. Initially set up in response to the observation that few Inuit were using the centre’s aftercare services and that many would eventually return to undertake another program, these activities proved to have other benefits with regards to cultural safety. For one thing, these staff journeys in the North led them to create connections that, while not entirely compensating for the geographical remoteness of residential programs, made it easier for Inuit residents currently in the centre programs to stay in contact with their community and relatives in the North. Another thing is that staff travelling to the North had the opportunity to have various experiences that contributed to improving their understanding of Inuit living conditions and the challenges they face, which helped these staff to better adjust their interactions and to put their interventions into perspective. Being able to see northern realities with one’s own eyes was the route to learning and knowledge most valued by both Inuit and staff.

#### Human resources and professional development

The centre can count on a workforce that is diverse in age, education, life experiences and, to a lesser extent, ethnocultural background. However, it did not have Inuit staff, which was perceived as a shortfall by Inuit residents and staff alike. Some shared their impression that Inuit staff could better understand the experiences and feelings of Inuit residents. For staff participants, having an Inuk on their team would help to bridge the gaps for mutual understanding and to deliver more responsive and effective programs to meet Inuit needs. Recruitment of Inuit staff was, however, a significant challenge for a number of reasons, including the limited pool of qualified people and the uncompetitive market in the South in comparison to northern employers. In the absence of Inuit staff, Inuit residents who were more advanced in the program were helping community members to separate what was more related to Inuit cultural practices or habits and what was not, thus contributing to resolving certain conflict situations through better understanding on both sides.

The centre could count on a non-Indigenous clinical advisor with a long experience in working with Indigenous peoples. This person was responsible for managing the centre’s special projects for Inuit in all their clinical, administrative and political aspects, as well as advising program managers on the development and delivery of services for First Nations people and Inuit. The advisor worked more closely with some of the centre’s staff, who would gain more experience working with Inuit. Most of the staff first arrived at the centre with little or no experience with Indigenous people and they learned on the job by working with the Inuit residents. While this form of knowledge transfer can help build relationships, dialogue, and trust over time, it represented a burden for many Inuit residents who may not have been willing or prepared to educate non-Inuit. Staff have expressed training needs related to the realities and living conditions of Inuit (especially in the North), their culture and ways of doing, and how to communicate effectively with them. Some staff were also interested in learning more about the history and success stories of Inuit to move beyond the negative portrayals generally found in the media and society, as well as knowing about resources available for them. From the perspective of staff with more experience working with Inuit, having some knowledge of the Inuit culture and a better understanding of historical issues was valuable in terms of stepping back, adjusting, and working with them better. This included intervening only on things related to the reasons that brought Inuit into the centre’s programs.

“I sincerely believe that we cannot ignore the historical trauma of Inuit. We have to understand the impact it has on the life of each individual we meet – because it is different, it’s not always the same. We have to take this into account on both individual and collective levels. It puts a lot of things into perspective. For staff, it helps to have some objective distance. Because often, when I encounter adversity – whether individually or in a group – I can more easily take an objective distance when I have this little box next to me that says, ‘Okay, that’s from his great-grand-father, so I’m going to put it in the box, it’s not between him and me, it’s between his People and mine.’ ” (Gérard).

Despite the obvious interest of many staff and the relevant experience accumulated by the centre, most of the training, support, and clinical supervision of workers regarding the Inuit context or cultural sensitivity was provided through informal meetings, either spontaneous or prompted by specific situations. This path of knowledge transfer was often based on personal initiative and was at times confronted with the closedness or resistance of some staff members to reconsider their practices.

### Systemic factors

More distal, the system-level factors considered here influenced the provision of programs deemed culturally safe by Inuit, through their effects on the individual, programmatic, and organizational factors previously described. We identified three predominant categories of systemic factors: (1) social climate; (2) relational, political, and funding climate; and (3) legislative, regulatory, and professional environment.

#### Social climate

For staff with experience working with Inuit, the interpersonal relationships of most Inuit residents at the centre, and thus their integration and participation in the therapeutic community, were greatly influenced by the social climate in the North. Staff spoke of a pervasive mistrust, exacerbated by the magnitude of addiction. The normalization of drinking and using and the lack of support to address them would create a climate conducive to social isolation of Inuit trying to overcome their problem, especially when returning from a treatment program in the South. This social climate seemed to explain, at least in part, the reluctance of Inuit residents to trust other residents in their program and the annoyance of many when they were told that they had to work with them rather than with staff. Besides, staff observed that Inuit residents seemed to share more openly about their experiences and feelings in the presence of non-Inuit staff or residents or in the context of one-on-one encounters.

Trust issues in the North also impacted relationships with non-Inuit. Some staff participants reported that alcohol or drug use by social service providers in the North undermined Inuit trust of them, already hampered by high staff turnover in these services and the fact that it was usually these same individuals who intervened to take children away from Inuit families when they lost custody.

“They’re [Inuit] suspicious of us [‘White’ people]. It’s like: ‘Yes, I want to help, yes… But after two years I’m going to leave…’ Because it happens, so they don’t invest themselves in relationships. I also learned that they are afraid to talk to the workers who are there, social workers and all that. Because they see some who are drunk. And they think: if I say something to her and she gets drunk, maybe she’ll talk about it…” (Laurent).

Falling more broadly within the various collective and individual trauma experienced by Inuit, some staff participants noted that the colonial or paternalistic aspect of the social intervention in the North could have a negative impact on the development of trusting relationships at the centre. This broader context in which service providers sometimes take matters into their own hands instead of letting Inuit handle it, was also likely to have a negative influence on Inuit residents’ satisfaction with the work of the centre staff when the latter were trying to promote their empowerment.

Within this social climate, the multiplicity of unsuccessful attempts to implement addiction interventions in the North was perceived by some staff as contributing to a sense of disillusionment about the support Inuit can expect to receive. This appeared to hamper the staff efforts to engage Inuit in designing the various activities that were offered to them in the South, as well as the aftercare support offered by the centre in the North.

#### Relational, political, and funding climate

Despite their influence on the centre’s ability to provide culturally adapted services to Inuit, political and funding issues were discussed with caution by staff, who inevitably emphasized their sensitive and volatile nature. Although the centre’s financial situation generally allows it to avoid service interruptions in its regular activities, offering a culturally specific and adapted programming to a subgroup of its program users was challenging as this required additional resources involving extra costs. The centre has sought and obtained the support of various Inuit and non-Inuit partners and funders to implement its special projects intended for Inuit. The sustainability of these specific projects remained dependent on the relational climate with partners and funders. Further development of the centre’s services for Inuit, notably a dedicated residential program for them, was also impeded by administrative, financial, and political considerations. One of the sensitivities was related to the desire for self-determination and control on the part of Indigenous communities and organizations over the delivery of public services to them.

“That’s complicated. Here you’re touching on political issues. You’re dealing with cultural issues. Everywhere in the country for… always, we’ll say, but especially for… about twenty years, you’ve had a desire for self-determination of First Nations and Inuit that’s been growing all the time. So, the more it goes, the more people want to do their own business. They want to do it at home. Which is perfectly legitimate.” (Gérard).

For staff participants, the emphasis on developing services *by* Inuit *for* Inuit led some Inuit organizations to refuse to collaborate with Southern ones. Staff participants regretted that this context limited the centre’s ability to continue the work it had begun, to develop trusting relationships with a growing number of Inuit and to set up projects to better support them. Staff participants also commented about the lack of resources dedicated to Inuit in Southern Quebec, especially places where they could safely refer Inuit residents for authorized outings during their program (i.e., places with no alcohol or drug use).

#### Legislative, regulatory, and professional environment

As a private institution with agreements with the Quebec Ministry of Health and Social Services, the centre is subject to various legislation, regulations, and professional standards that influence its service offer and its activities. Some of these elements would affect the capacity of the centre to better meet needs and expectations of Inuit residents. There were instances where the implementation of such rules or standards differentially affected Inuit residents because of their cultural practices or life circumstances, which led them to feel different and specially targeted.

The example most frequently cited by staff relates to the laws and regulations of the Quebec Ministry of Agriculture, Fisheries and Food (MAPAQ), which severely constrained the centre’s ability to serve traditional foods or allow them to be consumed on its premises. Since traditional foods were understood as important to most Inuit residents’ well-being, staff put in place a procedure to allow for the occasional consumption of traditional foods by Inuit residents over the course of their program, within certain guidelines. There were concerns, however, that the constraints imposed by the laws and regulations could contribute to the marginalization of Inuit residents and their practices.

“But we have limits, you know. Example: when they come with traditional foods, well I can’t put them in the dining room to eat that, because the MAPAQ doesn’t want to. […] But this, right off the bat, it isolates, you know. […] We have to live with it, and then we have to try to do it in a way that doesn’t make people feel restricted. That’s what the challenge is all about.” (Carl).

Another example was related to the over-representation of Inuit among residents with provincial judicial status and more limited cooperation of probation officers in the provincial court system due to shorter sentences. This situation created imbroglios in that Inuit sometimes felt that the constraints imposed on them by staff were specifically directed at them as Inuit rather than at any resident with (provincial) court orders imposed on them.

## Discussion

This study sought to explore the factors influencing the efforts of a mainstream residential addiction rehabilitation centre to provide culturally appropriate and quality care for Inuit. Participants talked extensively about individual- and program-level factors, focusing on the perceived and/or actual cultural differences between Inuit residents and non-Inuit staff and residents and the program. Staff also highlighted the growing experience of the centre of working with Inuit, the strengths of its model of care and areas where further adaptations could better accommodate Inuit residents, as well as the ways in which external contextual factors circumscribed their work. The results offer insight into the interplay between individual, programmatic, organizational, and systemic factors, acting as challenges and barriers that mainstream organization face when working with Inuit, and/or the opportunities and enablers they can build on to improve their services.

Building on the flexibility of the therapeutic approach and its person-centred orientation, several accommodations and adaptations have been made specifically to improve access, retention, completion, and continuity of care for Inuit program users. In this regard, staff participants emphasized the centre’s willingness and commitment to provide quality care to this population, especially through activities dedicated to Inuit residents where they could gather, eat traditional foods and meet with Elders. Inuit residents, however, rarely mentioned these efforts up front when asked about their experiences in the programs. This discrepancy could be partly explained by the fact that these activities may not be considered or understood by Inuit residents to be an integral part of the program and/or that they were too occasional to figure prominently in their overall experience. While many Inuit residents valued any opportunities to speak Inuktitut and to eat traditional food, others were not proficient in Inuktitut or less interested in traditional foods. Therefore, it cannot be assumed that these practices are universally shared by Inuit and that including them in programs would necessarily foster cultural safety. Several authors stress that the inclusion of Indigenous cultural practices in the programs, although welcomed [30, 31], would not be sufficient on its own to ensure that the programs are culturally safe and equitable [32–34]. Based on the work by Schill and Caxaj [35], strategies such as anticipating barriers to access and willingness to accommodate Indigenous practices would better be described as a cultural competency approach rather than a feature of cultural safety; the former being based on the development of “a set of congruent attitudes, behaviours, and policies […] to work effectively in cross-cultural situations” [36], while the latter explicitly focuses on self-awareness and reflexivity over power dynamics [14]. Also, focusing on cultural practices may contribute to a stereotypical view of Inuit and distract attention from making other significant changes to improve their experience in the programs [14, 34].

While the therapeutic community approach offered more time and opportunities to develop trustful relationships between individuals, it also brought a certain level of complexity when assessing the cultural safety of specific activities or interventions. For example, dedicated activities for Inuit were experienced as culturally safe when they happened, but at times prompted tensions due to negative reactions by some non-Inuit residents over what they perceived as a privilege for Inuit only. Conversely, staff requests of Inuit residents to separate from their Inuit peers were usually experienced as emotionally challenging and culturally unsafe at the moment, although by fostering the development of relationships with other residents, this situation generally increased inclusion of Inuit in the broader resident community in the longer term. These tensions need to be examined in the light of the broader historical and sociopolitical context in which they take place, given the pervasiveness of racism and discrimination in society and how it permeates all its systems, including health care [37–39]. Creating and maintaining a culturally safe environment thus require a special attention to, and consideration of how everyone’s power, privilege and bias are manifested and experienced on a daily basis within programs [14]. This includes, but is not limited to, addressing the ways in which a lack of awareness and knowledge of Indigenous realities as well as language barriers may influence relationships and decision-making within the therapeutic community and may have unintended, undesirable effects for some individuals or subgroups, for example by reinforcing prejudice and inequities. Similarly, attention should be given to the way in which the legislative, regulatory, and professional standards that organizations have to follow might affect the experiences and program outcomes of some of their program users differently, based on their distinctive ways of being or doing. The strategies used to resolve such tensions are critical to foster trust development, which also contributes to cultural safety [7, 11]. Ultimately, this comes down to the question of how focusing (or not) on a given subgroup of residents affects its cultural safety, and that of others in the group [40].

Other key challenges were related to aspects of staffing/human resources, professional development and partnerships, as interwoven with broader social, political, and economic forces. Despite having a diverse workforce, all programs relied on non-Inuit managers and staff, with only a few who had experience working with Inuit before they were hired. Overall, their experiences were consistent with and reflected different phases of the social psychological process described by McGough et al. [41] through which non-Indigenous mental health practitioners are able to overcome their experience of being unprepared to work with Indigenous people by seeking solutions to navigate the path of cultural safety. We also observed that their progression in this process was influenced by their own experiences of racism and discrimination, if any, and the level of support they received from peers, colleagues, and managers and by the organization [41]. In this regard, even though in-house expertise and resources were available, the fact that there was limited formal training or supervision relative to cultural safety and equity could interfere with further progress of staff and organization in these areas. This seems important, considering that many expressed support needs, and conversely others did not perceive the need or appeared reluctant to examine and change their practices.

To advance cultural safety, mainstream organizations are enjoined to engage Indigenous individuals or organizations in meaningful ways [8, 34, 35, 42]. A greater representation of Inuit can be achieved through the hiring of Inuit staff, patient navigators and/or Elders [8, 30, 43]. Depending on the situations and roles they are assigned to, these individuals may contribute to cultural safety in a number of ways, such as directly supporting and advocating on behalf of Inuit program users and their families; facilitating communication between Inuit and non-Inuit; and/or helping to educate and train non-Inuit staff about issues that may affect their care relationships [43]. In the absence of such actors, we observed that it was rather the Inuit residents who were more advanced in the programs who advocated, supported and bridged communications for newcomers, and educated non-Inuit about Inuit life context and culture. At the same time, there are documented challenges related to the recruitment and retention of Indigenous staff in health services, including in residential alcohol and drug treatment centres [22]. These challenges may be even greater when work expectations are not aligned with the family, social, and cultural responsibilities of Indigenous staff [20, 44], when their work may re-expose them to traumas they have experienced [34], and when there are concerns that their cultural identity, knowledge, and skills may not be recognized or respected in the workplace [45]. Each of these conditions adds to the fatigue, stress, and burnout that can be related to the working conditions and emotional demands associated with working in the field of addiction [19, 22]. All these elements reinforce the importance of offering all staff members adequate support to ensure their relational availability [8, 34].

On a different level, cultural safety encourages the empowerment and active participation of Indigenous people with respect to the planning, delivery, and evaluation of services intended for them [35]. Such participation and redistribution of power imply risks/threats, opportunities and responsibilities for both Indigenous and non-Indigenous parties, for mutual empowerment and equal partnerships [46]. Moving in this direction is prone to being politically charged and challenging, considering the legacies and potential replication of colonial practices that undermined Indigenous self-determination [21, 35]. It was therefore not surprising to observe that some Inuit and non-Inuit organizations had different perspectives of what cultural safety means and how to achieve it, all of which influenced their motivation and willingness to collaborate.

### Study limitations

The following limitations should be considered when reviewing this study. This work was conducted by a team of non-Indigenous academics. Even though the research methods allowed for a diversity of perspectives to be included in the study, the ones of the individuals most interested in the research topic are likely to be more prominent in the findings. These do not include the perspectives of senior management or the centre partners; this may have limited the scope of the analysis of the organizational and systemic factors. The transferability of study findings should be considered in light of the attributes of the selected case, including its model of care, the duration of its programs, and the characteristics of its staff and program users. Finally, we used reflexivity and perspective throughout the research process to minimize distortions in the way we represented Inuit and non-Inuit participants and organizations in the study.

## Conclusions

This study contributes to a better understanding of the factors that may influence the provision of culturally safe addiction rehabilitation programs to Inuit within a complex mainstream intervention setting. These factors reflect the combination of interrelated challenges and opportunities at multiple levels, that organizations must deal with to deliver responsive and quality care. By highlighting some areas for improvement, our work can help to guide interventions to advance cultural safety in this context. For example, it would be helpful to create spaces for collective reflection and constructive dialogue on elements that influence the relational dynamics and the development of trust. These include how bias, privilege, and power manifest and operate within programs and organizations, and ways to attend to resulting inequities [47]. Implementing or consolidating structures to provide tailored training and support to all staff, managers, and administrators in cultural safety and trauma and violence-informed care as part of equity-oriented care [48], as well as promoting organizational learning and ongoing knowledge transfer on these topics, can foster this reflective and dialogical process through the development of critical consciousness and awareness about the impacts of their own culture on the care they provide. Also, every effort must be made to engage Inuit individuals and organizations actively in the design, delivery, and evaluation of care, which entails taking into consideration the challenges, opportunities, and responsibilities of all parties involved in creating and maintaining equal and meaningful (i.e., beyond token) partnerships. Finally, systemic efforts are needed to enhance the training of Inuit and non-Inuit addiction workers, and to promote and support a variety of culturally safe addiction services for Inuit.

## Data Availability

The datasets generated and/or analyzed during the current study are not publicly available due to the confidential nature of the information they contain but are available from the corresponding author on reasonable request.

## Footnotes

2 In Canada, Inuit are one of three distinct First Peoples, along with First Nations and Métis. These three peoples are historically, culturally, and linguistically distinct from each other.

## References

[1] Gone JP, Trimble JE (2012). American Indian and Alaska Native mental health: diverse perspectives on enduring disparities. Annu Rev Clin Psychol.

[2] Adelson N. The embodiment of inequity: health disparities in Aboriginal Canada. Can J Public Health. 2005;96(Suppl 2):45–61. 10.1007/BF03403702 PMC6975716 16078555

[3] Chansonneuve D. Addictive Behaviours Among Aboriginal People in Canada. Ottawa: Aboriginal Healing Foundation; 2007 [cited 2021 Jan 12]. Available from: http://www.ahf.ca/downloads/addictive-behaviours.pdf.

[4] Bélanger RE, Muckle G, Courtemanche Y, Poliakova N. Substance use - Qanuilirpitaa? 2017 Nunavik Inuit Health Survey. Kuujjuaq, QC: Nunavik Regional Board of Health and Social Services; 2020 Nov [cited 2020 dec 4]. Available from: http://www.nrbhss.ca/en/nrbhss/public-health/health-surveys.

[5] Plourde C, Brunelle N, Landry M (2011). Alcohol and drug use in Nunavik: Converging views on the future.

[6] Horrill T, McMillan DE, Schultz ASH, Thompson G (2018). Understanding access to healthcare among Indigenous peoples: A comparative analysis of biomedical and postcolonial perspectives. Nurs Inq.

[7] Ramsden IM. Cultural Safety and Nursing Education in Aotearoa and Te Waipounamu [Doctoral thesis]. Wellington: Victoria University of Wellington; 2002.

[8] Cultural Safety Working Group. Holding Hope in Our Hearts: Relational Practice and Ethical Engagement in Mental Health and Addictions. First Nation Inuit and Métis Advisory Committee of the Mental Health Commission of Canada; 2013 [cited 2021 Jan 12]. Available from: http://www.mentalhealthcommission.ca/English/media/3227.

[9] Assembly of First Nations, National Native Addictions Partnership Foundation, Health Canada. Honouring Our Strengths: A Renewed Framework to Address Substance Use Issues Among First Nations People in Canada. Ottawa; 2011 [cited 2020 Dec 21]. Available from: http://nnadaprenewal.ca/wp-content/uploads/2012/01/Honouring-Our-Strengths-2011_Eng1.pdf.

[10] Wood PJ, Schwass M (1993). Cultural safety: a framework for changing attitudes. Nurs Prax N Z.

[11] Lauzière J, Fletcher C, Gaboury I. Cultural safety as an outcome of a dynamic relational process: The experience of Inuit in a mainstream residential addiction rehabilitation centre in Southern Canada. 2021. Manuscript submitted for publication. 10.1177/10497323221087540 PMC9189595 35350939

[12] Blanchet Garneau A, Pepin J. La sécurité culturelle: une analyse du concept. Rech Soins Infirm. 2012;(111):22–35. 23409542

[13] National Aboriginal Health Organization (2008). Cultural competency and safety: A guide for health care administrators, providers and educators.

[14] Curtis E, Jones R, Tipene-Leach D, Walker C, Loring B, Paine SJ (2019). Why cultural safety rather than cultural competency is required to achieve health equity: a literature review and recommended definition. Int J Equity Health.

[15] Reading C. Understanding racism. Prince George, BC: National Collaborating Centre for Aboriginal Health; 2013 [cited 2020 Dec 21]. Available from: https://www.nccih.ca/495/Understanding_racism.nccih?id=103.

[16] Browne A, Fiske J (2001). First Nations women’s encounters with mainstream health care services. West J Nurs Res.

[17] Cameron BL, Plazas MdPC, Salas AS, Bearskin RLB, Hungler K (2014). Understanding Inequalities in Access to Health Care Services for Aboriginal People. Adv Nurs Sci.

[18] Hole RD, Evans M, Berg LD, Bottorff JL, Dingwall C, Alexis C (2015). Visibility and Voice: Aboriginal People Experience Culturally Safe and Unsafe Health Care. Qual Health Res.

[19] Legha R, Raleigh-Cohn A, Fickenscher A, Novins D (2014). Challenges to providing quality substance abuse treatment services for American Indian and Alaska Native communities: perspectives of staff from 18 treatment centers. BMC Psychiatry.

[20] Taylor K, Thompson S, Davis R. Delivering culturally appropriate residential rehabilitation for urban Indigenous Australians: a review of the challenges and opportunities. Aust N Z J Public Health. 2010;34:36–40. 10.1111/j.1753-6405.2010.00551.x 20618291

[21] Taylor KP, Bessarab D, Hunter L, Thompson SC (2013). Aboriginal-mainstream partnerships: exploring the challenges and enhancers of a collaborative service arrangement for Aboriginal clients with substance use issues. BMC Health Serv Res.

[22] Chenhall RD, Senior K (2013). “The concepts are universal, it is the picture you paint that is different”: key issues for Indigenous Australian alcohol and drug residential treatment centres. Therapeutic Communities: The International Journal of Therapeutic Communities.

[23] Nilsen P (2015). Making sense of implementation theories, models and frameworks. Implement Sci.

[24] Stake RE (1995). The art of case study research.

[25] Abma TA, Stake RE (2014). Science of the Particular: An Advocacy of Naturalistic Case Study in Health Research. Qual Health Res.

[26] Creswell JW (2013). Qualitative inquiry & research design. Choosing among five approaches.

[27] National Institute on Drug Abuse (2015). Therapeutic communities.

[28] Thorne S (2016). Interpretative description: qualitative research for applied practice.

[29] Chaudoir SR, Dugan AG, Barr CH (2013). Measuring factors affecting implementation of health innovations: a systematic review of structural, organizational, provider, patient, and innovation level measures. Implement Sci.

[30] Bird CE, Thurston WE, Oelke N, Turner D, Christiansen K. Understanding cultural safety: traditional and client perspectives. Calgary: University of Calgary & Alpha House Society; 2013 Feb [cited 2021 Jan 12]. Available from: https://www.homelesshub.ca/resource/understanding-cultural-safety-traditional-and-client-perspectives.

[31] Freeman T, Edwards T, Baum F, Lawless A, Jolley G, Javanparast S, et al. Cultural respect strategies in Australian Aboriginal primary health care services: beyond education and training of practitioners. Aust N Z J Public Health. 2014;38(4):355–61. 10.1111/1753-6405.12231 25091076

[32] Maar MA, Erskine B, McGregor L, Larose TL, Sutherland ME, Graham D (2009). Innovations on a shoestring: A study of a collaborative community-based Aboriginal mental health service model in rural Canada. International Journal of Mental Health Systems.

[33] Oelke ND. A Participatory Case Study of Primary Healthcare for Aboriginal Peoples in an Urban Setting [Doctoral thesis]. Calgary: University of Calgary; 2010.

[34] Browne AJ, Varcoe C, Lavoie J, Smye V, Wong ST, Krause M (2016). Enhancing health care equity with Indigenous populations: evidence-based strategies from an ethnographic study. BMC Health Serv Res.

[35] Schill K, Caxaj S (2019). Cultural safety strategies for rural Indigenous palliative care: a scoping review. BMC Palliat Care.

[36] Cross TL, Bazron BJ, Dennis KW, Isaacs MR (1989). Towards a Culturally Competent System of Care: A Monograph on Effective Services for Minority Children Who Are Severely Emotionally Disturbed.

[37] Browne AJ (2017). Moving beyond description: Closing the health equity gap by redressing racism impacting Indigenous populations. Soc Sci Med.

[38] Paradies Y, Harris R, Anderson I (2008). The impact of racism on Indigenous health in Australia and Aotearoa: towards a research agenda.

[39] Allan B, Smylie J. First Peoples, second class treatment: The role of racism in the health and well-being of Indigenous peoples in Canada. Toronto, ON: the Wellesley Institute; 2015 Feb [cited 2021 Jan 12]. Available from: http://www.wellesleyinstitute.com/publications/first-peoples-second-class-treatment/.

[40] Arieli D, Hirschfeld MJ (2013). Supporting minority nursing students: ‘Opportunity for Success’ for Ethiopian immigrants in Israel. Int Nurs Rev.

[41] McGough S, Wynaden D, Wright M. Experience of providing cultural safety in mental health to Aboriginal patients: A grounded theory study. Int J Ment Health Nurs. 2018;27(1):204-13. 10.1111/inm.12310 28165178

[42] Berry SL, Crowe TP (2009). A review of engagement of Indigenous Australians within mental health and substance abuse services. Australian e-Journal for the Advancement of Mental Health.

[43] Foster P. Champions of cultural safety: an exploration of how cultural safety can be implemented as a routine aspect of health care [Master thesis]. Vancouver: University of British Columbia; 2017.

[44] Roche AM, Duraisingam V, Trifonoff A, Battams S, Freeman T, Tovell A, et al. Sharing stories: Indigenous alcohol and other drug workers’ well-being, stress and burnout. Drug Alcohol Rev. 2013;32(5):527–35. 10.1111/dar.12053 23675893

[45] Deroy S, Schutze H (2019). Factors supporting retention of aboriginal health and wellbeing staff in Aboriginal health services: a comprehensive review of the literature. Int J Equity Health.

[46] Brascoupé S, Waters C. Cultural Safety - Exploring the Applicability of the Concept of Cultural Safety to Aboriginal Health and Community Wellness. J Aborig Health. 2009;5(1–3):6–41.

[47] Nixon SA (2019). The coin model of privilege and critical allyship: implications for health. BMC Public Health.

[48] Browne AJ, Varcoe C, Ford-Gilboe M, Nadine Wathen C, Smye V, Jackson BE (2018). Disruption as opportunity: Impacts of an organizational health equity intervention in primary care clinics. Int J Equity Health.

